# Elucidating the grading intricacies of idiopathic multicentric Castleman disease histopathology: a pathologist’s perspective

**DOI:** 10.1093/ajcp/aqaf134

**Published:** 2026-03-03

**Authors:** Daisy V Alapat, Payam Etebari, Frits Van Rhee

**Affiliations:** Department of Pathology, University of Arkansas for Medical Sciences, Little Rock, AR 72205, United States; Recordati Rare Diseases, Bridgewater, NJ, United States; Myeloma Center, University of Arkansas for Medical Sciences, Little Rock, AR, United States

**Keywords:** idiopathic multicentric Castleman disease, histopathology, grading, diagnosis

## Abstract

**Objective:**

Idiopathic multicentric Castleman disease (iMCD) is a rare condition with a wide range of signs and symptoms that often overlap with other conditions that cause lymphadenopathy. The diagnosis of iMCD remains challenging, even among experts. Our aim is to highlight the heterogeneity of histopathologic features and presentations of iMCD and to provide tools to help diagnose and raise awareness of this rare condition.

**Methods:**

We review the most relevant clinicopathologic aspects of iMCD and present our process to accurately diagnose this condition using a case-based approach. We describe how to incorporate the iMCD diagnostic criteria, including grading of histomorphology, laboratory, and clinical findings.

**Results:**

Here, we describe our system to grade histopathologic features in iMCD. Together with radiologic findings and laboratory and clinical information, this system helps clinicians accurately identify iMCD and its subtypes, leading to more appropriate and timely diagnosis and treatment.

**Conclusions:**

The diagnosis of iMCD can be challenging and requires a multidisciplinary team of hematologists, pathologists/hematopathologists, and other specialists. There is a pressing and unmet clinical need for a harmonized system to grade the histopathologic features of iMCD. This condition should be included in the differential diagnosis when other causes of a lymphoproliferative disorder (eg, infections, autoimmune/inflammatory disorders, malignancy) have been ruled out.

KEY POINTS

- The diagnosis of iMCD can be challenging due to disease presentations that often overlap with other causes of lymphadenopathy as well as a lack of specific disease biomarkers.
- We report a harmonized method that builds on previous knowledge to quantitatively grade the histopathologic features of iMCD and help clinicians accurately diagnose this rare condition.
- Recognizing and distinguishing iMCD from other causes of lymphadenopathy are fundamental to guide appropriate clinical management.

## INTRODUCTION

**Figure 1:**
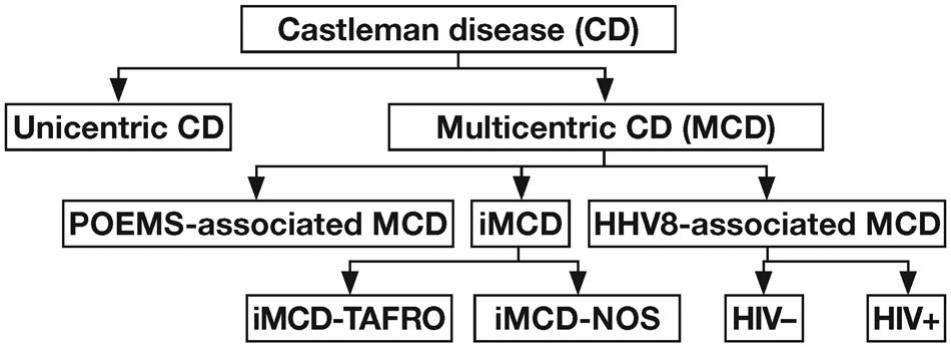
Classification of Castleman disease. CD indicates Castleman disease; HHV8, human herpesvirus 8; iMCD, idiopathic multicentric Castleman disease; iMCD-NOS, idiopathic multicentric Castleman disease, not otherwise specified; iMCD-TAFRO, idiopathic multicentric Castleman disease with thrombocytopenia, anasarca, fever, renal insufficiency/kidney dysfunction, and organomegaly; MCD, multicentric Castleman disease; POEMS, polyneuropathy, organomegaly, endocrinopathy, monoclonal plasma cell disorder, skin changes.

Castleman disease (CD, formerly referred to as angiofollicular lymph node hyperplasia) is a rare condition first described by Benjamin Castleman in 1954 as a localized mediastinal lymph node mass in otherwise asymptomatic patients.^1^^,^^2^ Subsequent reports identified histopathologic variants of CD, which ranged from hyaline vascular to plasma cell, as well as cases with mixed histopathology that incorporates features of hyaline vascular and plasma cell types.^3^^,^^4^ This condition is primarily classified according to the extent of lymph node involvement as unicentric CD (involving a single lymph node station) and multicentric CD (MCD; involving ≥2 lymph node stations) (Figure 1).^4^ Most patients with unicentric CD are asymptomatic and amenable to surgery, whereas patients with MCD usually have constitutional symptoms (fever, night sweats, weight loss, and fatigue) and laboratory abnormalities (anemia, elevated C-reactive protein [CRP] and erythrocyte sedimentation rate, hypergammaglobulinemia, thrombocytosis or thrombocytopenia, hypoalbuminemia) that wax and wane over the clinical course of the disease.^5^ Multicentric CD is further subdivided by etiology as human herpesvirus 8 (HHV-8)–associated MCD (HHV-8–MCD); polyneuropathy, organomegaly, endocrinopathy, monoclonal plasma cell disorder, skin changes (POEMS)–associated MCD (POEMS-MCD); and idiopathic MCD (iMCD, also called HHV-8–negative MCD). Though the etiology of iMCD is unknown, interleukin 6 (IL-6) has been identified as a key pathologic contributor.^6^^,^^7^ The idiopathic subtype represents approximately one-third to one-half of all MCD cases, with clinical presentations that can vary from mild to life-threatening disease and a 5-year mortality rate of 35% (before the approval of anti–IL-6 therapies).^4^^,^^8^ Most patients with iMCD have a not-otherwise-specified form (iMCD-NOS), but a small subset of patients present with a severe form of iMCD characterized by thrombocytopenia, anasarca/ascites, fever, reticulin fibrosis in bone marrow/kidney dysfunction, and organomegaly (iMCD-TAFRO)^6^^,^^9^^,^^10^

Evidence-based consensus guidelines on the diagnosis and treatment of iMCD were published in 2017 and 2018.^11^^,^^12^ Despite the availability of diagnostic guidelines and CD-specific *International Statistical Classification of Diseases* codes, the diagnosis of iMCD remains challenging, even among experts, due to the variability of clinical and histopathologic features. This is illustrated in an analysis conducted by 3 independent panels of expert hematopathologists, where consensus on the histopathologic subtype of iMCD (*n* = 79) was achieved in only 23% of cases.^13^ Additional hurdles to diagnosis include disease presentations and histopathologic similarities that often overlap with other causes of lymphadenopathy as well as a lack of specific disease biomarkers. For these reasons, it takes a multidisciplinary team of hematologists, pathologists/hematopathologists, and other specialists to accurately diagnose iMCD. In this report, we review the most relevant aspects of iMCD and present our process to diagnose this condition using a case-based approach. We describe how to incorporate the iMCD diagnostic criteria, focusing on grading of histomorphology, laboratory, and clinical findings. Our objective is to highlight the pathologist’s perspective of the heterogeneity of histopathologic features of iMCD and to provide tools to accurately diagnose and raise awareness of this rare condition.

## DISTINGUISHING HYALINE VASCULAR, PLASMA CELL, AND MIXED HISTOPATHOLOGY

The involvement pattern of affected lymph nodes can be heterogeneous, so an excisional lymph node biopsy is required to evaluate the histopathologic variant within the iMCD spectrum (from hypervascular type to plasma cell type).^11^^,^^14^ Excisional lymph node biopsy allows for examination of complete nodal architecture, unlike fine needle aspiration or needle core biopsy, which often fail to yield good quality samples for a definitive CD diagnosis.^14^ These nonpreferred types of biopsies, however, may be warranted in special cases (eg, difficult access to affected lymph nodes, high risk of bleeding, urgency).

The pathologist primarily reviews the lymph node biopsy to identify and grade histopathologic features based on the presence of regressed or hyperplastic germinal centers, follicular dendritic cell (FDC) prominence, vascularity, and plasmacytosis.^11^ Briefly, a hyaline vascular histopathologic subtype is characterized by the presence of regressed germinal centers, FDC prominence, and hypervascularity, whereas a plasma cell subtype commonly displays hyperplastic germinal centers and profuse interfollicular plasmacytosis. The mixed subtype of iMCD includes a combination of both.

### Hyaline vascular/hypervascular

The hyaline vascular subtype refers to an atypical lymphoid proliferation with unique morphologic features in the lymphoid follicles and interfollicular areas. Lymphoid follicles typically show expanded mantle zones of small mature lymphocytes concentrically arranged around 1 or more atretic germinal centers in an “onion skin” pattern (Figure 2A). Vascularity appears to be prominent in the germinal centers as well as in interfollicular areas, often with perivascular hyalinization (Figure 2B). The concentric layering of mantle zone lymphocytes, together with a prominent germinal center–penetrating hyalinized central vessel, provides the appearance of a “lollipop” (Figure 2C). In some cases, follicular and interfollicular areas show prominent fibrosis and hyaline deposits, which may artificially show features such as “cracking artifacts” on tissue sections (Figure 2D). Immunohistochemically expanded mantle zone B cells can show dim variable positivity for CD5 in addition to CD20 and BCL2 expression, and the B lymphoid compartment is classically polytypic and typically identified by flow cytometry.^15^ Regressed follicles have lymphoid depletion, sometimes reveal dysplastic FDCs (Figure 2E) with an expanded FDC meshwork, and some follicles show features of “twinning” (Figure 2F).^16^ The expanded interfollicular areas are composed of small mature lymphocytes (predominantly T cells), prominent vascular proliferation with hyalinization (including high endothelial venules [HEVs]), myoid cells, fibroblastic reticulum cells (occasionally with atypical nuclear features), and small aggregates of plasmacytoid DCs.

**Figure:**
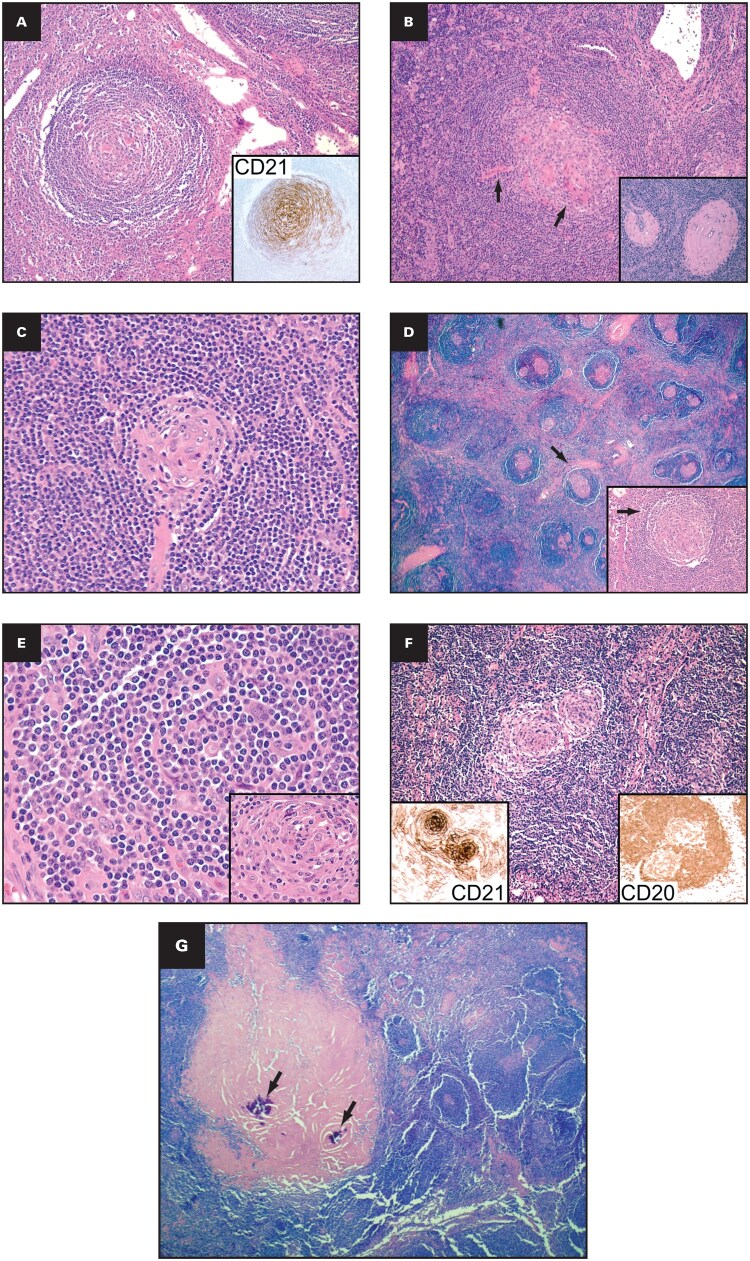
**Figure 2** Histopathologic features of hyaline vascular/hypervascular iMCD. **(A)** Follicles have widened mantle zones with concentric arrangement of mantle zone cells or an onion skin pattern; HE stain in 20x magnification (inset: CD21 immunostain highlighting FDC mesh works; in 40x magnification); **(B)** vascular hyperplasia and perivascular hyalinization; HE stain in 20X magnification (arrow and inset; HE stain in 20x magnification); **(C)** atrophic germinal centers with penetrating hyalinized central vessel provides the appearance of a lollipop (HE stain in 40x magnification); **(D)** follicles with features of “cracking artifact”, HE stain in 4x Magnification (arrow; inset follicle with cracking artifact; HE stain in 40x magnification); **(E)** dysplastic FDC cells (inset HE stain in 100x magnification); **(F)** follicles with features of “twinning” HE stain in 20x magnification (insets: Immunostains -CD21 and CD20 twinning; in 20x magnification); **(G)** lymph node with prominent fibrosis and granulomas associated with calcification (HE stain in 10x magnification). HE, hematoxylin and eosin; FDC ­indicates follicular dendritic cell; iMCD, idiopathic multicentric Castleman disease.

In some cases, the interfollicular stromal component is prominent, including clinically significant fibrosis, calcifications, and granulomas (Figure 2G). Plasma cells and eosinophils are not prominent, and sinusoids are typically closed. Vasculitis is not observed. Indolent T-lymphoblastic proliferation with a common thymocyte stage of cortical thymocyte differentiation that lacks monoclonality in the interfollicular regions has also been described in some cases. Rare reports of clonal cytogenetic abnormalities in CD21-positive FDC involving *HMGIC* have also been described.^17^ Another report suggests that the hyaline vascular subtype of CD is a monoclonal process, most likely originating from stromal cells, with some cases displaying conventional genetic abnormalities.^18^ There are also reports of FDC sarcomas and vascular neoplasms in some patients with this histopathologic variant of iMCD.^19^ In iMCD-TAFRO, the lymph node vascularity is so prominent (without associated, prominent hyalinization) that it can be described as hypervascular rather than hyaline vascular.^20^

### Plasma cell/plasmacytic

The plasma cell/plasmacytic histopathologic variant typically involves multiple lymph nodes, which radiologically and clinically present as a mass. In the majority of cases, the lymph node architecture is preserved, showing variably sized follicles with a predominance of hyperplastic follicles (Figure 3A). The FDC network appears to be normal, with a minimal mantle zone hyperplasia and prominent interfollicular plasmacytosis (Figure 3B). The plasma cells are typically polytypic and have mature morphology. In POEMS-MCD, the interfollicular plasma cell aggregates are characteristically immunoglobulin A (IgA) λ light chain restricted. Another characteristic feature is the presence of plasmacytoid immunoblasts (plasmablasts) in the mantle zone or paracortical regions (Figure 3C). Vascular hyperplasia is also seen in this variant, and open dilated sinuses are frequently observed. It is important to highlight that these morphologic features are nonspecific: They can be associated with other entities, such as chronic infections, autoimmune disorders (collagen vascular and mixed connective tissue disease, rheumatoid arthritis), Castleman type IgG4-related lymphadenopathy, immunodeficiency-associated lymphadenopathy, and malignancies. Exclusion of these reactive states is of paramount importance before establishing a diagnosis of plasma cell/plasmacytic CD.

**Figure:**
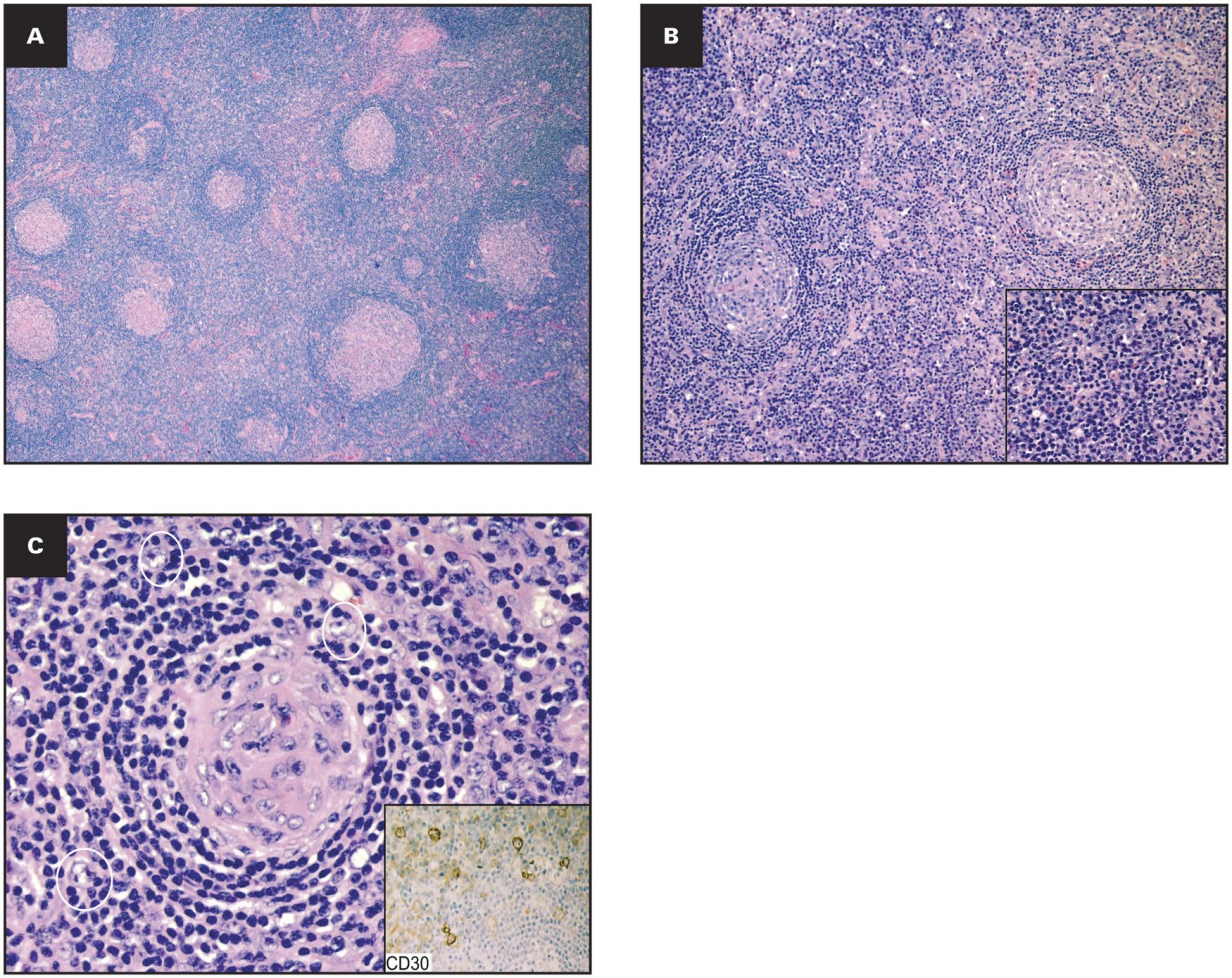
**Figure 3** Histopathologic features of plasma cell/plasmacytic idiopathic multicentric Castleman disease. **(A)** Hyperplastic follicles HE stain in 4x magnification; **(B)** prominent interfollicular plasmacytosis; HE stain in 10x magnification (inset: HE stain higher magnification- 40x); **(C)** presence of plasmacytoid immunoblasts “white circles” HE in 40x magnification (inset: Immunostain CD30-positive immunoblasts in 40x magnification). HE, hematoxylin and eosin.

### Mixed histopathology

**Figure 4:**
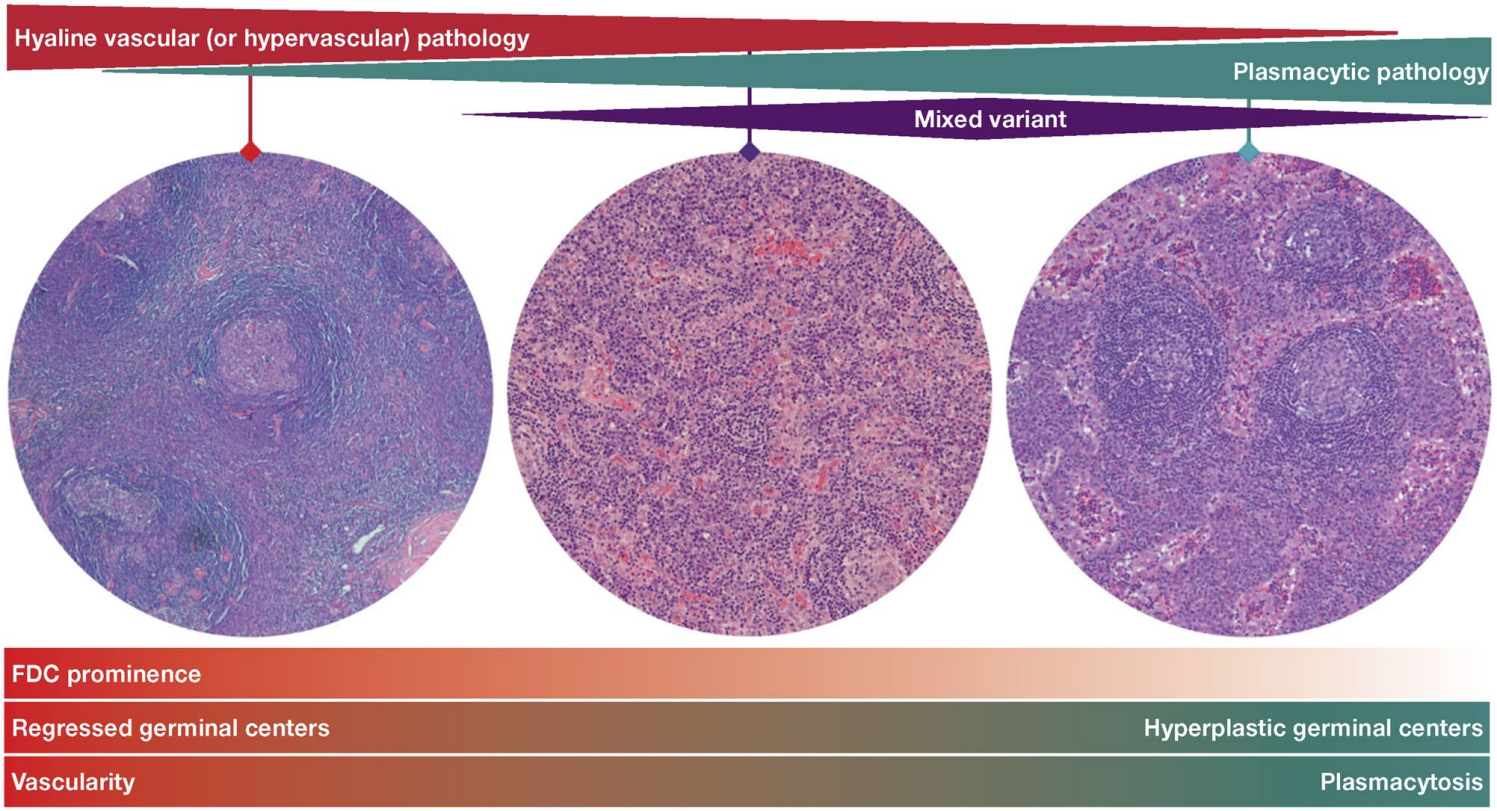
Histopathologic spectrum of idiopathic multicentric Castleman disease. FDC indicates follicular dendritic cell.

The histopathology of a mixed variant exhibits both hyaline vascular and plasma cell features (Figure 4). The coexistence of morphological features of both subtypes within the same lymph node or in different lymph nodes in the same patient may represent transition states within the histopathologic spectrum of this condition.

## DIAGNOSTIC CRITERIA OF iMCD

In 2017, Fajgenbaum et al.^1^ introduced the Castleman Disease Collaborative Network consensus diagnostic criteria for the diagnosis of iMCD, which include 2 major criteria and 11 minor criteria (Table 1). To diagnose iMCD, cases must meet both major criteria and at least 2 minor criteria, of which at least 1 should be a laboratory abnormality. These consensus criteria also require active exclusion of other conditions, which can give rise to similar symptoms and histopathology (infections, autoimmune/autoinflammatory diseases, malignant/lymphoproliferative disorders [LPDs]). Clinically significant bone marrow findings include reticulin fibrosis (in the TAFRO type) and reactive, polyclonal plasmacytosis.

**Table 1:** Major and Minor Criteria for Diagnosing iMCD

Major criteria (both required)	Minor criteria (need ≥2, with ≥1 laboratory criterion)
Histopathologic lymph node features consistent with the iMCD spectrum (need grade 2-3 for either regressive germinal centers or plasmacytosis at minimum): Regressed germinal centers^a^ FDC prominence Vascularity^b^ Plasmacytosis^c^ Hyperplastic germinal centers Multicentric lymphadenopathy (≥2 lymph node stations)^d^	Clinical criteria: Constitutional symptoms^e^ Skin changes^f^ Enlarged spleen and/or liver Fluid accumulation Pulmonary involvement Laboratory criteria: Elevated CRP (>10 mg/L) or erythrocyte sedimentation rate (>15 mm/h)^g^ Polyclonal hypergammaglobulinemia (total γ globulin or IgG > 1700 mg/dL) Anemia (hemoglobin <12.5 g/dL for males, <11.5 g/dL for females) Kidney dysfunction (estimated glomerular filtration rate <60 mL/min/1.73 m^2^) or proteinuria (total protein = 150 mg/24 h or 10 mg/100 mL) Thrombocytosis (>400k/μL) or thrombocytopenia (<150k/μL) Hypoalbuminemia (<3.5 g/dL)

Abbreviations: CRP, C-reactive protein; FDC, follicular dendritic cell; Ig, immunoglobulin; iMCD, idiopathic multicentric Castleman disease.

a Often with expanded mantle zones composed of concentric rings of lymphocytes in an “onion skin” appearance.

b Often with prominent endothelium in the interfollicular space and vessels penetrating into the germinal centers with a “lollipop” appearance.

c Sheetlike, polytypic plasmacytosis in the interfollicular space.

d Enlarged lymph nodes defined as ≥1 cm in short-axis diameter.

e Night sweats, fever (>38 °C), weight loss, or fatigue (Common Terminology Criteria for Adverse Events grade ≥2 lymphoma score for B symptoms).

f Eruptive cherry hemangiomatosis or violaceous papules. A specific skin feature called cutaneous plasmacytosis (multiple violaceous cutaneous plaques with numerous polyclonal plasma cells) is commonly observed in patients of Asian ethnicity.

g Evaluation of CRP is mandatory, and tracking CRP levels is highly recommended; however, erythrocyte sedimentation rate will be accepted if CRP is not available.

This research was originally published in *Blood* online. Fajgenbaum DC et al. International, evidence-based consensus diagnostic criteria for HHV-8–negative/idiopathic multicentric Castleman disease. Published March 23, 2017. doi:10.1182/blood-2016-10-746933.^11^

### Grading histologic features of iMCD

**Figure 5:**
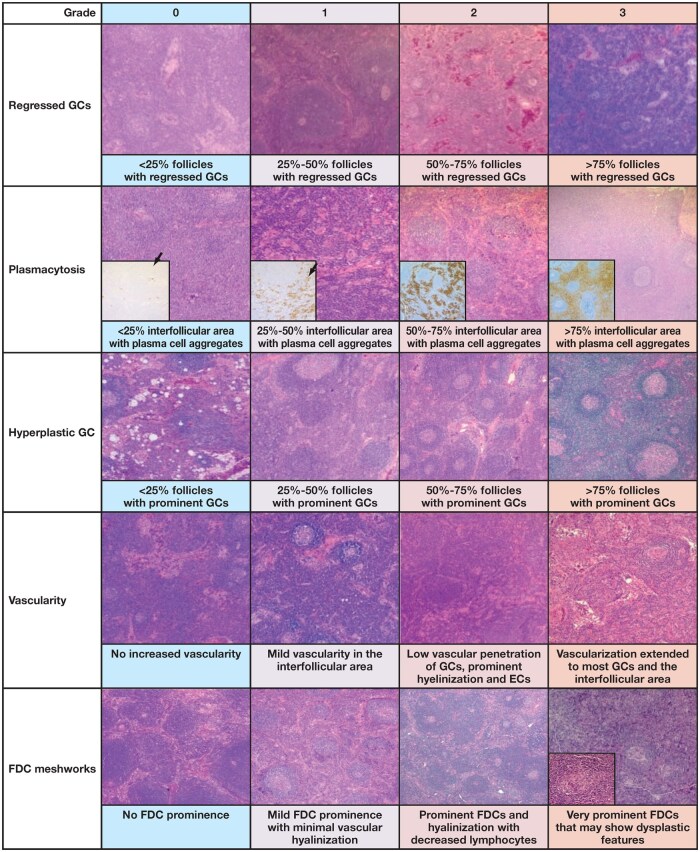
Histomorphologic grading of iMCD features. EC indicates endothelial cell; FDC, follicular dendritic cell; GC, germinal center; iMCD, idiopathic multicentric Castleman disease.

To satisfy the major criteria, patients need histopathology grade 2 or higher regressed germinal centers or plasmacytosis in addition to multicentric lymphadenopathy (lymph node size should be ≥2 cm). Although the other histologic features described in Table 1 are not necessary, the presence of these features can support the diagnosis. Criteria for grading each histologic feature are not described distinctly in the literature. To grade the histologic features of iMCD, a method based on a grading system used for follicular lymphoma has been employed.^21^ This system adds supplemental quantitative data to the one previously reported by Fajgenbaum et al.^11^ Below, we describe our methodology for grading the histologic features of iMCD (Figure 5).

#### Regressed germinal centers

Regressed germinal centers with expanded mantle zones are composed of concentric rings of lymphocytes with an onion skin appearance. The onion skin pattern, however, does not need to display a perfectly concentric ring of lymphocytes, and irregular circular formats are acceptable, as well. To be considered expanded, mantle zone thickness should be more than one-third of the size of the germinal center, with relative layering and no features of polarization. It is important to highlight that the size of the mantle zone of each lymphoid follicle varies based on age, location in the body, immune status, and the presence of other conditions. Characteristics of atrophic germinal centers include thick, concentric mantle zones; nonreactive marginal zones; lack of reactive features within the germinal centers (no or rare tingible body macrophages, small numbers of centroblasts, no germinal center polarization [segregation of the centroblast and centrocytes into pale and dark zones]); hyalinized blood vessels penetrating the atrophic germinal centers, providing a “lollipop” feature; hyaline deposits in atrophic germinal centers; lymphocyte depletion in germinal centers; and contracted FDC mesh works highlighted by CD21, with the extension of the FDC processes into the surrounding thick mantle zones. Normal lymph nodes have few regressed germinal centers, whereas in iMCD, these nodes tend to present different degrees of regression. For grading purposes, we define regressed follicles as typically smaller than normal (appearing as shrunken or underdeveloped), with a lack of reactive features or markedly decreased reactivity and increased FDCs, which may be penetrated by hyalinized vessels. The grading system for this histologic feature is based on the proportion of regressed follicles in lymph nodes (%) as grade 0 (< 25%), grade 1 (25%-50%), grade 2 (50%-75%), and grade 3 (>75%). In our assessment, grade 2 to 3 germinal center regression can be present without radially penetrating vessels or “twinning.”

#### Grading of plasmacytosis

Plasmacytosis grading is defined by the proportion of the interfollicular area replaced by plasma cells, with grade 0 being few plasma cells (either single or in small groups). The proportion of plasma cell aggregates found in the interfollicular space is mildly increased in grade 1 (25%-50%) and progressively larger in grade 2 (50%-75%) and grade 3 (>75%) (Figure 5). These plasma cells typically appear mature and are polytypic.

#### Hyperplastic germinal centers

Hyperplastic germinal centers are more prominent than normal germinal centers and occupy a larger section of the lymphoid follicles, encompassing more than 50% of the follicle. In CD, however, germinal center polarization is not typically seen in hyperplastic germinal centers. Enlarged FDC mesh works are highlighted by CD21 and confined to germinal centers, which contain an admixture of centrocytes, centroblasts, a few scattered plasma cells, and small T lymphocytes. The grading of this histologic feature is based on the proportion of follicles with prominent germinal centers (%). This proportion increases gradually from grade 0 (rare/absent hyperplastic germinal centers) and grade 1 (<25%) to grade 2 (50%-75%) and grade 3 (>75%) (Figure 5).

#### Grading of vascularity

Grading of vascularity considers the extent and location of vascular proliferation within the lymph node (which often penetrates the germinal center in a lollipop appearance) as well as the degree of vascular hyalinization and prominence of endothelial cells. We consider vascular hyalinization to be present when the hyalinized portion is more than one-third the thickness of the vessel wall. Prominent endothelial cells include tall, plump cells that form the lining of HEVs (located in the paracortex and T-cell zones of the lymph nodes) and lymphatic endothelial cells (located in subcapsular, cortical, and medullary sinuses). Although HEVs a have thick basal lamina and concentrically arranged fibroblasts (perivascular sheath), lymphatic endothelial cells in CD typically reveal sinus obliteration. We consider endothelial prominence to be present when the majority (>50%) of the vessels under observation have features of HEVs with hyalinization. In grade 0, lymph nodes do not show increased vascularity in the follicular and interfollicular areas, with no prominent endothelial cells. Vascularity appears to be mildly increased in grade 1 and is mostly prominent in the interfollicular area. In grade 2, vascularity is moderately increased, with low penetration of germinal centers. Most vessels display hyalinized vascular walls and prominent endothelial cells. This vascular proliferation extends to the majority of germinal centers and the interfollicular area in grade 3 (Figure 5).

#### FDC prominence

The prominence of FDCs is defined based on the number of FDCs present within the lymphoid follicles. The grading of FDC prominence includes grade 0 (no FDC prominence in >75% of follicles), grade 1 (mild FDC prominence, where FDCs replace 25%-50% of each follicle in 25%-50% of follicles in the lymph node sections), grade 2 (moderate FDC prominence with a decreased number of lymphocytes, where FDCs replace 50%-75% of germinal center cellularity in 50%-75% of follicles), and grade 3 (prominent FDCs that may show dysplastic features, where >75% of each follicle is replaced by FDCs in >75% of follicles) (Figure 5). This grading system factors in the degree of vascular hyalinization in the germinal centers to differentiate grade 1 (vascular hyalinization is minimal) from grade 2 (vascular hyalinization is prominent).

### Excluding diseases that mimic iMCD

**Figure 6:**
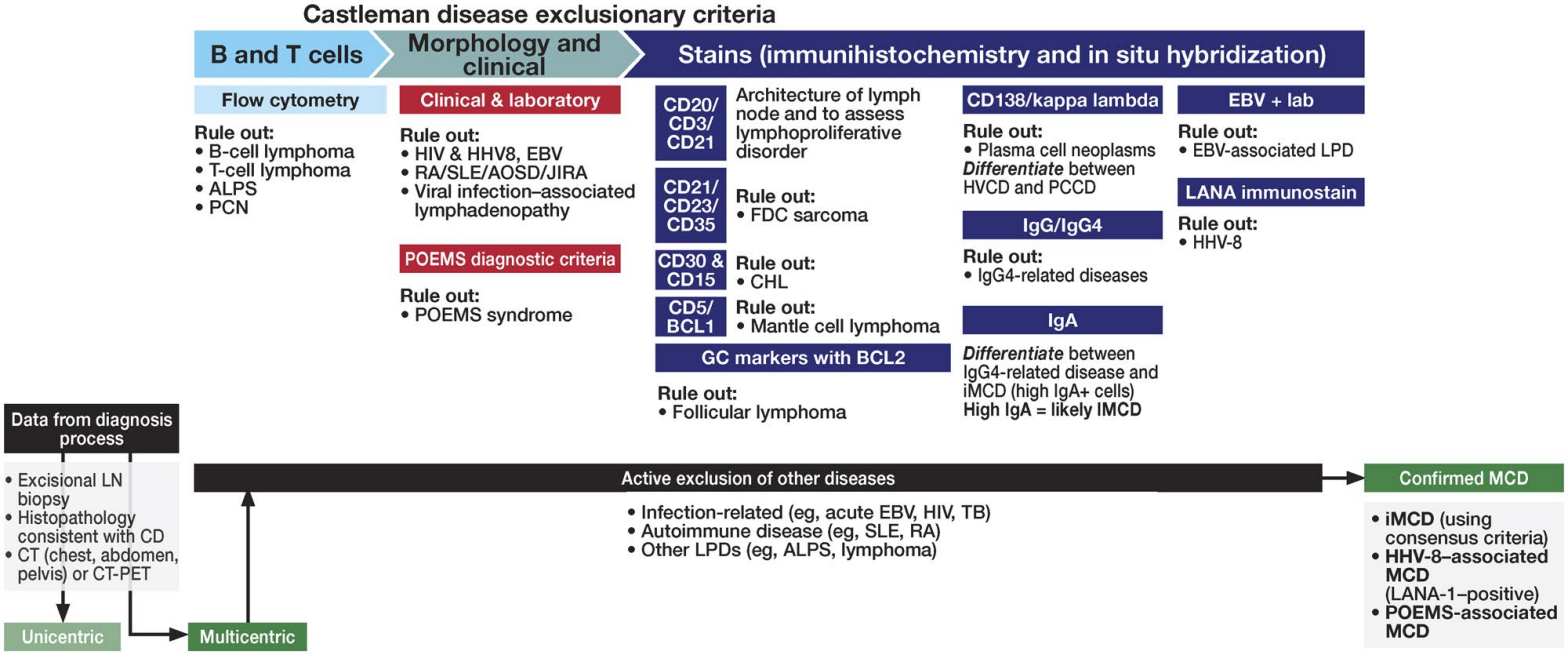
Workup to exclude iMCD-mimicking disorders. ALPS indicates autoimmune lymphoproliferative disease; AOSD, adult-onset Still disease; BCL2, B-cell lymphoma-2; CD, Castleman disease; CHL, classic Hodgkin lymphoma; CT, computed tomography; CT-PET, computed tomography–positron emission tomography; EBV, Epstein-Barr virus; FDC, follicular dendritic cell; GC, germinal center; HHV8, human herpesvirus 8; HVCD, hyaline vascular Castleman disease; Ig, immunoglobulin; iMCD, idiopathic multicentric Castleman disease; JIRA, juvenile idiopathic rheumatoid arthritis; LANA-1, latency-associated nuclear antigen 1; LN, lymph node; LPD, lymphoproliferative disease; MCD, multicentric Castleman disease; PCCD, plasma cell Castleman disease; PCN, plasma cell neoplasms; POEMS, polyneuropathy, organomegaly, endocrinopathy, monoclonal plasma cell disorder, skin changes; RA, rheumatoid arthritis; SLE, systemic lupus erythematous; TB, tuberculosis.

Idiopathic MCD encompasses clinical, histopathologic, and immunologic features that overlap with multiple malignant, infectious, and autoimmune/inflammatory conditions.^11^ As part of the consensus criteria for establishing a diagnosis of iMCD, patients should meet the exclusion criteria detailed in Table 2. Considering the clinical heterogeneity of iMCD and the differences in treatment among similarly presenting conditions, excluding diseases with overlapping signs or symptoms is essential.^11^^,^^22^ In addition to excisional lymph node biopsy, the workup for iMCD may involve blood work to exclude infections; measure inflammatory markers, cytokines, and autoantibodies; and assess organ function, imaging, and bone marrow evaluation, with ancillary flow cytometric and molecular studies.^11^^,^^12^ In certain scenarios, additional biopsies may also be necessary when prior needle core or excisional biopsies do not represent the diagnostic lesion. Any relevant findings of the workup should be interpreted in the context of the greater clinical picture. This workup is described in detail in Figure 6.

**Table 2:** Exclusion Criteria for Diagnosis of iMCD^a,11^

Infection-related disorders	Autoimmune/inflammatory disorders	Malignant/LPDs
HHV-8 (latency-associated nuclear antigen, immunohistochemistry, and blood polymerase chain reaction) Clinical EBV- LPDs (eg, acute/chronic EBV infection) (EBV, in situ hybridization, and polymerase chain reaction) Inflammation and adenopathy caused by uncontrolled infections (eg, acute or uncontrolled cytomegalovirus, toxoplasmosis, HIV, active tuberculosis) (provided clinical information, morphologic changes with immunohistochemistry and polymerase chain reaction) Viral hemophagocytic lymphohistiocytosis	SLE (morphology: cellular necrosis, vasculitis with necrosis, hematoxylin bodies; if ANA positivity is <1:80, it is unlikely to be SLE) Rheumatoid arthritis (clinical information with joint pain, necrosis) Adult-onset Still disease, consistently increased ferritin level >1000 ng/mL, low titer ANA and RF Juvenile idiopathic arthritis Autoimmune lymphoproliferative syndrome (increased double-negative T cells by flow cytometry, immunohistochemistry) IgG4-related disease (CD lymphadenopathy can show increased IgG4 positive cells, but if IgA-positive plasma cells are high, CD lymphadenopathy precedes over IgG4-related LPD	Classic Hodgkin lymphoma (immunohistochemistry—CD30) Non-Hodgkin lymphoma (flow cytometry/immunohistochemistry) Peripheral T-cell lymphoma (flow cytometry/immunohistochemistry) Multiple myeloma (clinical, radiologic, and laboratory findings) Primary lymph node plasmacytoma (immunohistochemistry) FDC sarcoma (morphology and immunohistochemistry) POEMS syndrome (meeting diagnostic criteria)^b^ Malignancy-associated hemophagocytic lymphohistiocytosis Lymphadenopathy associated with metastatic carcinomas

Abbreviations: ANA, antinuclear antibody; CD, Castleman disease; EBV, Epstein-Barr virus; FDC, follicular dendritic cell; HHV-8 ,  human herpesvirus 8; IgG4, immunoglobulin G4; iMCD, idiopathic multicentric Castleman disease; LPD, lymphoproliferative disorder; POEMS, polyneuropathy, organomegaly, endocrinopathy, monoclonal plasma cell disorder, skin changes; RF, rheumatoid factor; SLE, systemic lupus erythematosus.

a Must all be excluded.

b POEMS is considered a disease “associated” with CD. Because monoclonal plasma cells are believed to drive the cytokine storm, POEMS should not be considered iMCD but rather POEMS-associated iMCD.

This research was originally published in *Blood* online. Fajgenbaum DC et al. International, evidence-based consensus diagnostic criteria for HHV-8–negative/idiopathic multicentric Castleman disease. Published March 23, 2017. doi:10.1182/blood-2016-10-746933.^11^

#### Autoimmune/inflammatory disorders

Castleman-like histopathology is often observed in patients with autoimmune disorders. For instance, an estimate of 15% to 29% of patients with systemic lupus erythematosus (SLE) and most patients with rheumatoid arthritis had lymph node histopathology consistent with CD.^11^^,^^23‐25^ In contrast, autoimmune antibodies commonly found in autoimmune disorders can also be detected in some patients with iMCD.^11^^,^^22^ A systematic review of data collected from 128 patients with iMCD found that approximately 30% were positive for at least 1 type of autoimmune antibody (including antinuclear antibodies [ANAs]), positive Coombs test, antiplatelet antibodies, rheumatoid factor [RF], and anti–Ro/SS-A) or reported autoimmune hemolytic anemia.^26^ The ANAs are often nonspecific and of low titer (<1:80). Therefore, the presence of autoimmune antibodies is not enough to rule out iMCD, and patients must also meet full clinical and laboratory criteria for the suspected autoimmune/inflammatory disorder.^11^ In these cases, the differential may require additional laboratory workup in addition to clinical information.

Some patients with iMCD show increased IgG4-positive plasma cells with associated fibrosis. In this scenario, performing immunohistochemistry stains for other heavy chains (eg, IgM, IgD, IgA), CD30, and programmed cell death 1 protein (PD-1) is recommended.^27^ The presence of abundant IgA-positive plasma cells in the lymph node is suggestive of iMCD rather than IgG4-related disease, while IgG4-related LPDs have increased follicular T helper cells (PD-1), increased CD30-positive immunoblasts in the lymph nodes, and elevated serum IL-4 and IL-21 levels in addition to increased eosinophils or obliterative phlebitis. In addition, levels of laboratory markers such as CRP, IL-6, and immunoglobulins (IgA and IgM) are higher in iMCD compared with IgG4-related disorders. Expert consensus has determined that even with very high serum levels of IgG4, a diagnosis of iMCD should displace the diagnosis of IgG4-related disease.^11^

#### Malignancies/LPDs

Patients with iMCD have a 3-fold increased rate of malignancy (19%) compared with age-matched controls (6%).^26^ The exact causes of this correlation are not yet fully understood, but concurrent malignancies (diagnosed before, during, or shortly after the iMCD diagnosis) may lead to a proinflammatory cytokine storm that mimics the classic features of iMCD. For this reason, diagnoses of lymphoma (Hodgkin and non-Hodgkin), primary lymph node plasmacytoma, FDC sarcoma, or POEMS syndrome before or at the same time as iMCD are exclusionary.^11^ In contrast, hematologic malignancies occurring more than 1 year after iMCD diagnosis should not reverse the iMCD diagnosis.

Differentiating malignancies from iMCD can be difficult because both may display CD-like histopathology. Some examples include classic Hodgkin lymphoma with an interfollicular distribution pattern or FDC sarcoma with features of hyaline vascular histopathology (ie, dysplastic FDCs). In these cases, the presence of such features is not sufficient to establish a solid diagnosis of iMCD; however, morphologic features such as the presence of CD30-positive Reed-Sternberg–type cells in the interfollicular area are suggestive of classic Hodgkin lymphoma, and the prominence of at least 1 FDC marker (CD21, CD23, CD35) and dysplastic FDC cells suggest FDC sarcoma. It should be noted that in the hyaline vascular CD subtype, dysplastic FDC cells can be observed in atretic germinal centers. Finally, FDC sarcoma and hyaline vascular CD can be related: *N666S* mutation can occur in both hyaline vascular CD and FDC sarcoma.^28^ A bone marrow biopsy is recommended to differentiate suspected iMCD from MCD-POEMS.^11^ The absence of monoclonal protein with monotypic plasma cells in the bone marrow, polyneuropathy, and endocrine disorders can be used to exclude POEMS syndrome.^29^^,^^30^

#### Infectious diseases

Lymphadenopathy associated with viral illness, especially Epstein-Barr virus (EBV), HHV-8, and HIV infections, can show morphologic features similar to those observed in patients with iMCD. The classification of MCD subtypes is based on HHV-8 status.^12^ This viral agent is the most common etiologic agent of MCD and is associated with increased viral IL-6 in patients with HHV-8–MCD, which can be ruled out by testing the peripheral blood for HHV-8 by quantitative polymerase chain reaction. These patients tend to present with features of MCD and associated Kaposi sarcoma involving the same lymph node. Serology may merely reflect past exposure to HHV-8, and associated MCD should not be used to diagnose HHV-8.^11^^,^^12^ Alternatively, the lymph node biopsy can be stained for the latency-associated nuclear antigen 1 of the HHV-8 virus. Compromised immunity is a major risk factor for HHV-8–MCD, and most cases occur in patients who are positive for HIV^6^; EBV-related disorders can be diagnosed using in situ hybridization for EBV-encoded RNA or similar stains.

### Common scenarios when diagnosing iMCD

A definitive iMCD diagnosis should meet major criteria, minor criteria, and exclusion criteria (Tables 1 and 2).^11^ Patients who meet the major and exclusion criteria but do not meet the minor criteria are classified as probable for iMCD. Pathologists may not receive classic clinical pictures or laboratory results; in addition, histopathology is not unique and can be associated with other disorders. Clinicians should evaluate and order appropriate testing to rule out Castleman lymphadenopathy. Because the evaluation of lymph node architecture is important for the diagnosis of CD, another prominent issue pathologists commonly encounter is the lack of appropriate amounts of tissue for analysis. This issue is common with core needle biopsies and fine needle aspirates, which may reveal only fragments of lymphoid tissue composed of the paracortical area, rare follicles, primary follicles, or fibrotic tissue, all of which are insufficient for diagnosis. An intact excisional lymph node biopsy is required to make a definitive diagnosis.

### How do we differentiate iMCD-TAFRO from iMCD-NOS?

Further subclassification into iMCD-TAFRO and iMCD-NOS is recommended.^6^ For iMCD-TAFRO, diagnosis requires characteristic lymph node histopathology negative for HHV-8, the presence of 4 clinical criteria (thrombocytopenia, anasarca, fever or hyperinflammatory status [CRP ≥2.0 mg/dL], and organomegaly), and at least 1 additional criterion (renal insufficiency/kidney failure or TAFRO-consistent bone marrow, with reticulin fibrosis or megakaryocyte hyperplasia).^30^ Supportive clinical criteria (not required for diagnosis) include the absence of hypergammaglobulinemia and elevated alkaline phosphatase without markedly elevated transaminases.

Although both types of iMCD can show thrombocytopenia, patients with iMCD-NOS can also have a thrombocytosis or normal platelet count.^30^^,^^31^ When anasarca is present, patients with iMCD-NOS typically have milder effusion than those with iMCD-TAFRO. Fever and features associated with the inflammatory process are more prominent in iMCD-TAFRO. Bone marrow findings in patients with iMCD-TAFRO typically include reticulin fibrosis with megakaryocytic hyperplasia, whereas in patients with iMCD-NOS, the most common finding is mild polytypic plasmacytosis, with an adequate amount of megakaryocytes and unremarkable morphology without reticulin fibrosis. Both subtypes show organomegaly, but it is usually more prominent in iMCD-TAFRO. Serum immunoglobulin levels are relatively normal or mildly increased in iMCD-TAFRO compared with marked increases seen in iMCD-NOS. In iMCD-TAFRO, vascular endothelial growth factor levels are often high, which, together with severe hypoalbuminemia, can cause anasarca. Both subtypes can display mixed histopathology, with the hypervascular type being more common in iMCD-TAFRO and plasma cell/mixed types being observed more often in iMCD-NOS.

## EXAMPLES OF OUR CASE-BASED APPROACH

### Case 1: iMCD-NOS

A 50-year-old Asian man presented with cough, shortness of breath during exercise, weight loss, night sweats, and diminished energy. Computed tomography (CT) scans at that time revealed mediastinal lymphadenopathy, and he underwent bronchoscopy, with biopsy showing nonspecific changes. This finding prompted mediastinoscopy with excisional biopsy, which was interpreted as polyclonal plasmacytosis. His symptoms persisted, and a year later, he came to our institution. At that time, his laboratory findings showed a normal complete blood cell count, an albumin level of 2.5 g/dL, elevated inflammatory markers (CRP = 48.8 mg/L, erythrocyte sedimentation rate = 39 mm/h), and increased IL-6 (67 pg/mL). The patient had pan-hypergammaglobulinemia, with elevated IgG (3380 mg/L), IgA (579 mg/L), IgM (649 mg/L), κ (13.7), and λ (8.39). The κ:λ ratio was normal, and immunofixation was negative for a monoclonal protein. Molecular and serum immunology studies for HIV, EBV, and HHV-8 were negative; ANA and RF were negative. Positron emission tomography/CT scans showed extensive supraclavicular axillary, mediastinal, and hilar lymphadenopathy, with the largest lymph node measuring 7.4 mm and having a maximum standard uptake value of 4.2. Lymphadenopathy was also identified in the below-diaphragm area, with multiple mesenteric nodes, multiple small retroperitoneal nodes, multiple external iliac nodes, and bilateral inguinal nodes. The radiologic study was clinically significant for multilobar peribronchial ground-glass opacities radiating from the hilum, predominantly involving the upper lobes, middle lobes, and lingula, with relative sparing of the lung bases. A repeat mediastinal lymph node biopsy was performed. The patient’s lymph node had few regressed germinal centers (25% of total), mild FDC prominence, and moderately increased vascularity; most (>75%) of the germinal centers were hyperplastic, with sheet-like interfollicular plasmacytosis. Based on the lymph node histology grading system for CD described above, our assessment for the lymph node histopathology findings was grade 1 germinal center atrophy, grade 1 FDC prominence, grade 2 vascularity, grade 2 germinal center hyperplasia, and grade 3 plasmacytosis (Figure 7A and B). In some areas, lymphoid follicles were less well defined, with dissolution manifested by blurring of the boundary between the mantle zones and the surrounding interfollicular areas. The interfollicular areas and medulla were also expanded by large sheets of mature plasma cells (Figure 7C). Vascular proliferation with patent sinuses and histiocytic aggregates were also observed (Figure 7D). Immunohistochemistry with CD20 and CD3 stains showed intact immunoarchitecture, with the follicles composed primarily of CD20-positive B cells and the paracortex composed mainly of CD3-positive T cells (Figure 7E and F). CD138 stain and in situ hybridization studies of κ and λ light chains showed expanded interfollicular polytypic plasma cell aggregates (Figure 7G-I). Viral panels with EBV-encoded RNA and HHV-8 immunostain were negative. Concurrent flow cytometry analysis showed no immunophenotypic evidence of B-cell or T-cell LPD or clonal plasma cell neoplasm. The morphologic features of the lymph node were consistent with involvement by the plasma cell variant of CD. Bone marrow findings were clinically significant for mild hypercellularity, with trilineage maturation without prominent plasmacytosis. Based on the international consensus, diagnostic criteria for HHV-8–negative iMCD, the morphologic findings of the lymph node, laboratory findings, and the clinical findings met the requirements of major, minor, and exclusion criteria for diagnosis of iMCD (Tables 1 and 2). The patient was enrolled in a clinical trial with siltuximab and achieved remission. Seven years later, commercial siltuximab was discontinued due to loss of insurance, and he relapsed within 9 months with B symptoms, abnormal laboratory findings, and extensive fluorodeoxyglucose-avid lymphadenopathy. Siltuximab was restarted, and remission was recaptured. The patient is presently doing well, 5 years later, on siltuximab 11 mg/kg every 3 weeks.

**Figure:**
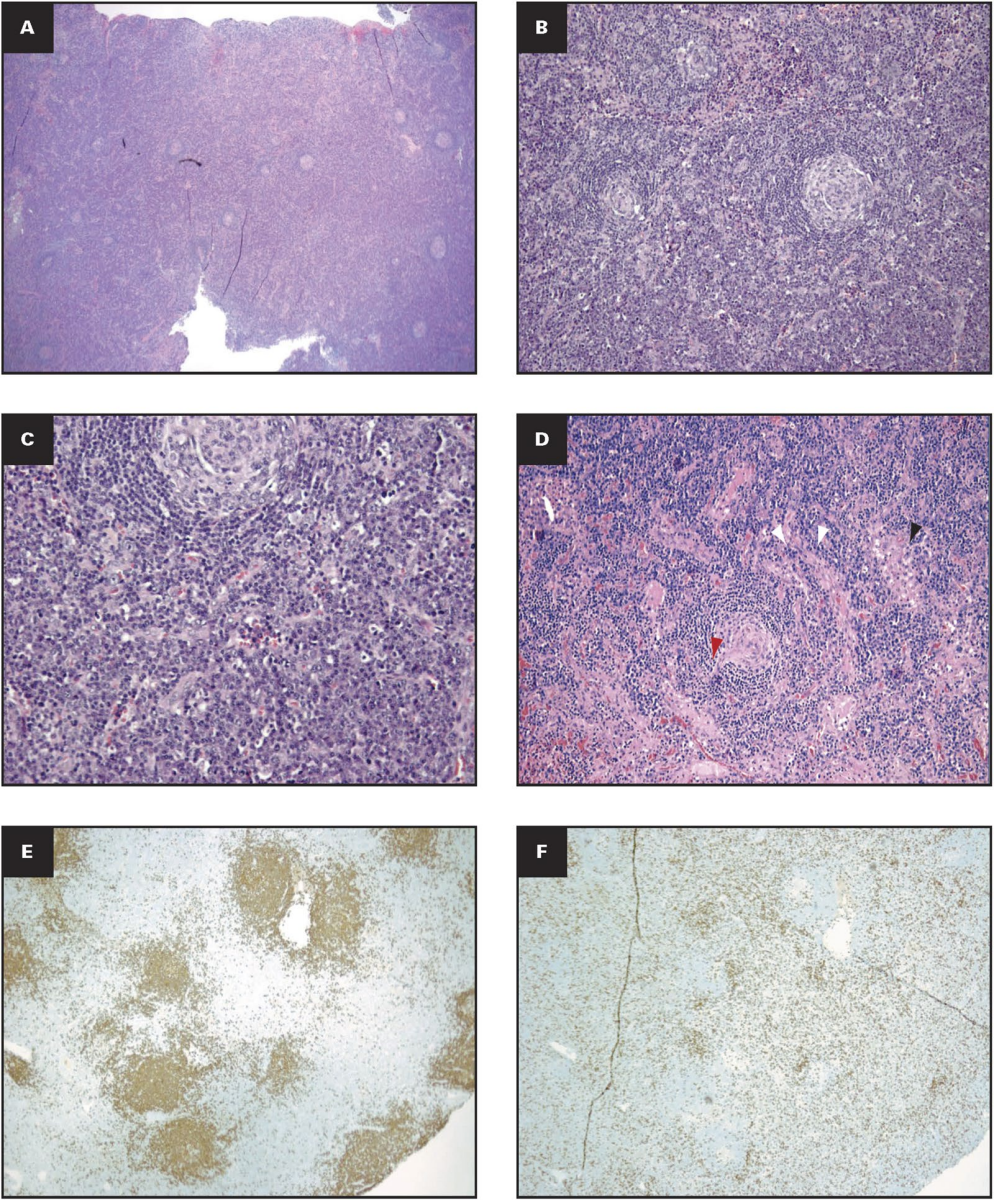

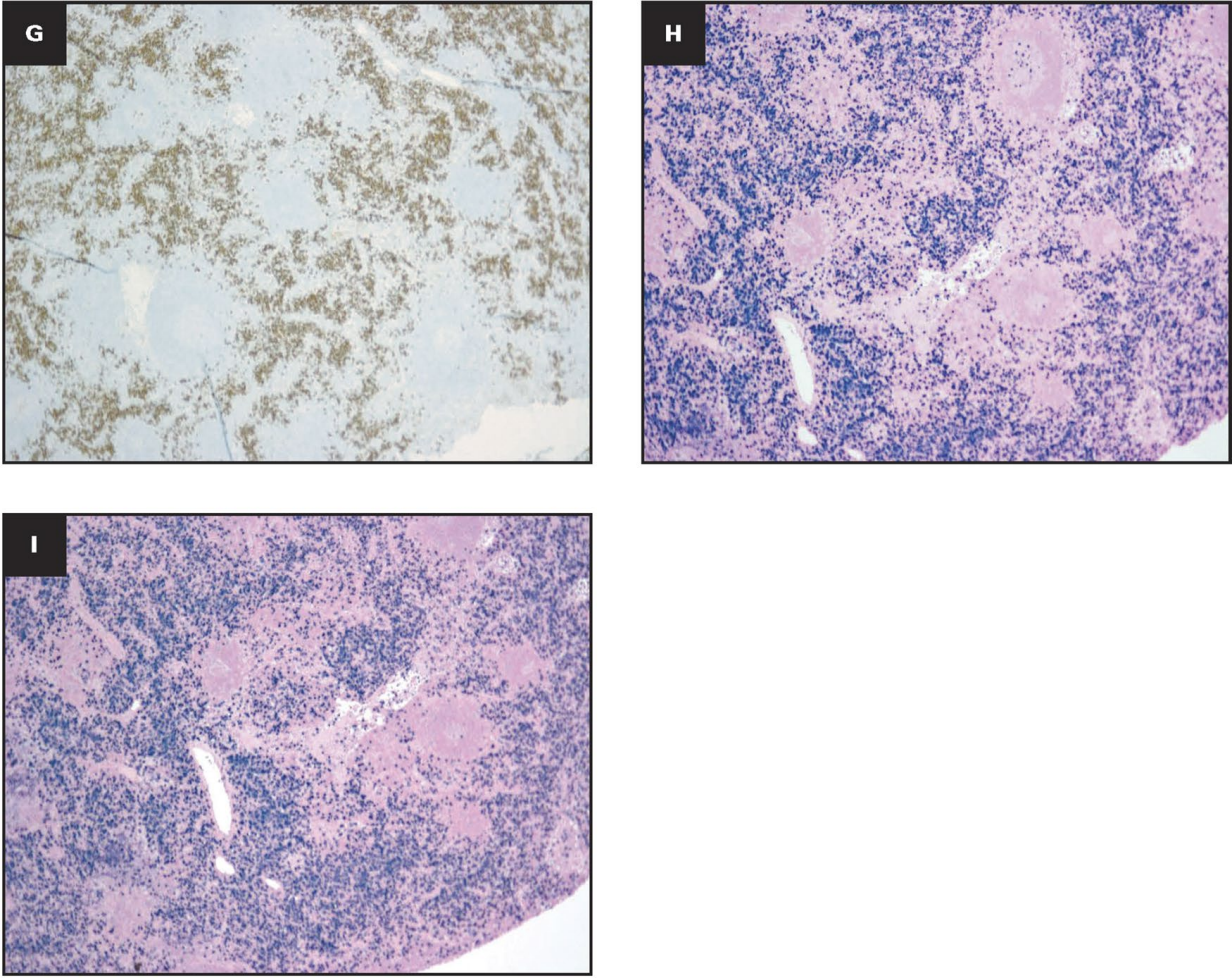
**Figure 7** Histopathologic characteristics of a cervical lymph node from excisional biopsy. Case 1: **(A)** Lymph node with abnormal architecture with polymorphic follicles, with a predominance of hyperplastic germinal centers; HE stain in 2x magnification (grade 2 hyperplastic follicles); **(B)** germinal centers with expanded mantle zones, with concentric layering and hyaline deposits; HE stain in 10x magnification (grade 2 follicular dendritic cell prominence); **(C)** interfollicular areas with large sheets of plasma cells (grade 3 plasmocytosis), HE stain in 40x magnification; **(D)** lymph node highlighting increased vascularity (white arrowhead), regressed follicles with hyalinized vessel–penetrating germinal center; HE stain in 10x magnification (red arrowhead), open sinuses with histiocytes (black arrowhead); **(E)** Immunostain CD20-positive B cells highlighting germinal centers in 4x magnification; **(F)** Immunostain CD3-positive T lymphocytes in 4x magnification; **(G)** Immunostain CD138-positive interfollicular plasma cells in 2x magnification; **(H, I)** κ and λ light chains (in situ hybridization) in 2x magnification. HE, hematoxylin and eosin.

### Case 2: iMCD-TAFRO

A 24-year-old woman with a medical history of anxiety and depression and unremarkable for physical illness presented to an outside hospital after 1 month of ongoing nausea, vomiting, diarrhea, fever, and weight loss with abdominal pain, night sweats, and chills. She was admitted to the family medicine service for evaluation of thrombocytopenia (platelet count = 27 ×10^9^/L), anemia (hemoglobin = 9.9 g/dL), colitis, and a urinary tract infection. A CT scan revealed diffuse thickening of the colon, reactive nodes in the mesentery, shotty pericardiac nodes, portal edema of the liver, and borderline hepatosplenomegaly. A chest x-ray showed bilateral pleural effusions with minimal volume ascites, and a chest CT showed lymph node enlargement (bilateral axillary, mediastinal, and hilar) without evidence of pulmonary embolism. Laboratory findings were clinically significant for increased CRP (17.23 mg/dL), positive lupus anticoagulant, and elevated dimerized plasmin fragment D. Autoimmune (ANA, RF, anti–cyclic citrullinated peptide, and anticentromere antibodies), infectious disease (viral panel, tickborne disease panel, fungal and bacterial panels, ova, and parasite), and gastrointestinal workup (celiac disease panel) were all negative. Nephrology was consulted for recurrent anasarca and acute kidney injury (estimated glomerular filtration rate = 47 mL/min, proteinuria). The oncology team was consulted for cytopenias, and bone marrow biopsy findings were clinically significant for diffuse moderate fibrosis and megakaryocytic hyperplasia. Because most of the lymph nodes were small and the patient was in poor physical condition, a fine needle aspirate biopsy of the cervical lymph node was performed, and the findings were unremarkable. The combination of laboratory and radiologic findings in the context of the clinical picture was concerning for a systemic inflammatory process such as MCD. The patient was transferred to our hospital for further evaluation, definitive diagnosis, and further treatment.

Bone marrow biopsy slides from the outside hospital were reviewed at our institution and showed 50% cellular bone marrow with megakaryocytic hyperplasia and dysplasia in the background of diffuse moderate fibrosis (Figure 8A-C). An excisional biopsy of an inguinal lymph node was performed, and the histologic sections showed a lymph node with distorted follicular and interfollicular architecture. Most follicles were atretic and scattered throughout the cortex and medulla (Figure 8D). The regressively transformed germinal centers had lymphoid depletion, focal deposition of hyaline material (Figure 8E), and vascular proliferation, often with penetrating hyalinized vessels, providing a lollipop morphologic pattern. Rare follicles had widened mantle zones composed of concentric rings of small mature lymphocytes. The interfollicular regions were depleted of lymphocytes and composed of numerous HEVs with plump endothelial cells, with a subset showing sclerotic/hyalinized walls ­(Figure 8F). Interfollicular plasma cell aggregates were minimally increased. CD20 immunostain showed an atretic nature of the follicles without a prominent expansion of the mantle zone (Figure 8G). The slight prominence of paracortical and interfollicular/intrafollicular T cells was observed with the CD3 immunostain (Figure 8H). The atretic nature of the follicles was also observed with the CD21 immunostain, highlighting the FDC mesh works. Small aggregates of polytypic plasma cells in the interfollicular area were highlighted by CD138 immunostain and κ and λ in situ hybridization (Figure 8I-K). Viral molecular studies and immunostains for EBV and HHV-8 were negative. Most of the plasma cells were IgG, with rare scattered IgG4 expression (<2% of IgG plasma cells). Based on the lymph node histopathology grading system for CD described above, our assessment of the lymph node histopathology findings was grade 3 germinal center atrophy, grade 3 FDC prominence, grade 3 vascularity, grade 0 germinal center hyperplasia, and grade 1 plasmacytosis. A concurrent flow cytometry study showed no immunophenotypic evidence of B-cell or T-cell LPD.

**Figure:**
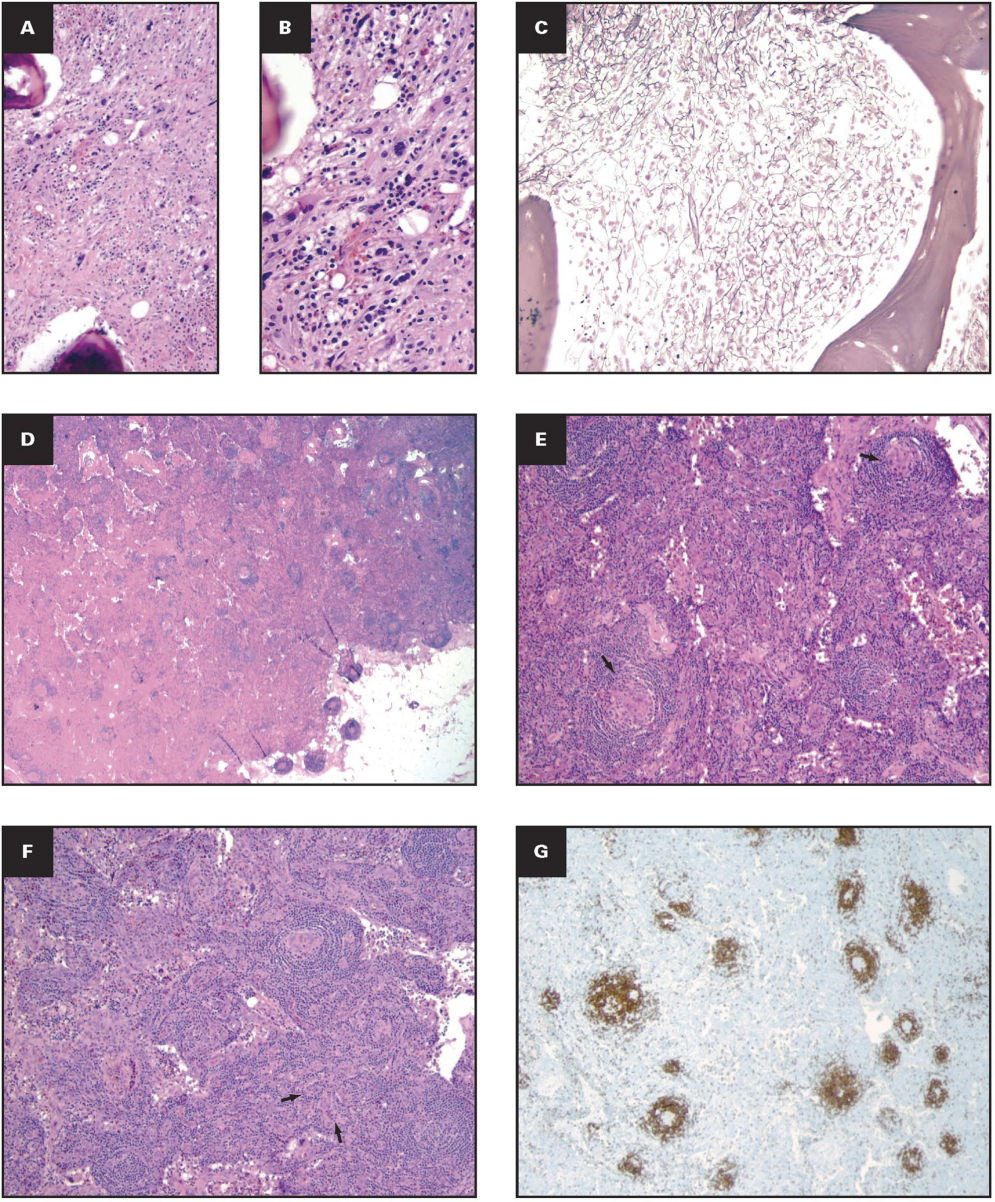

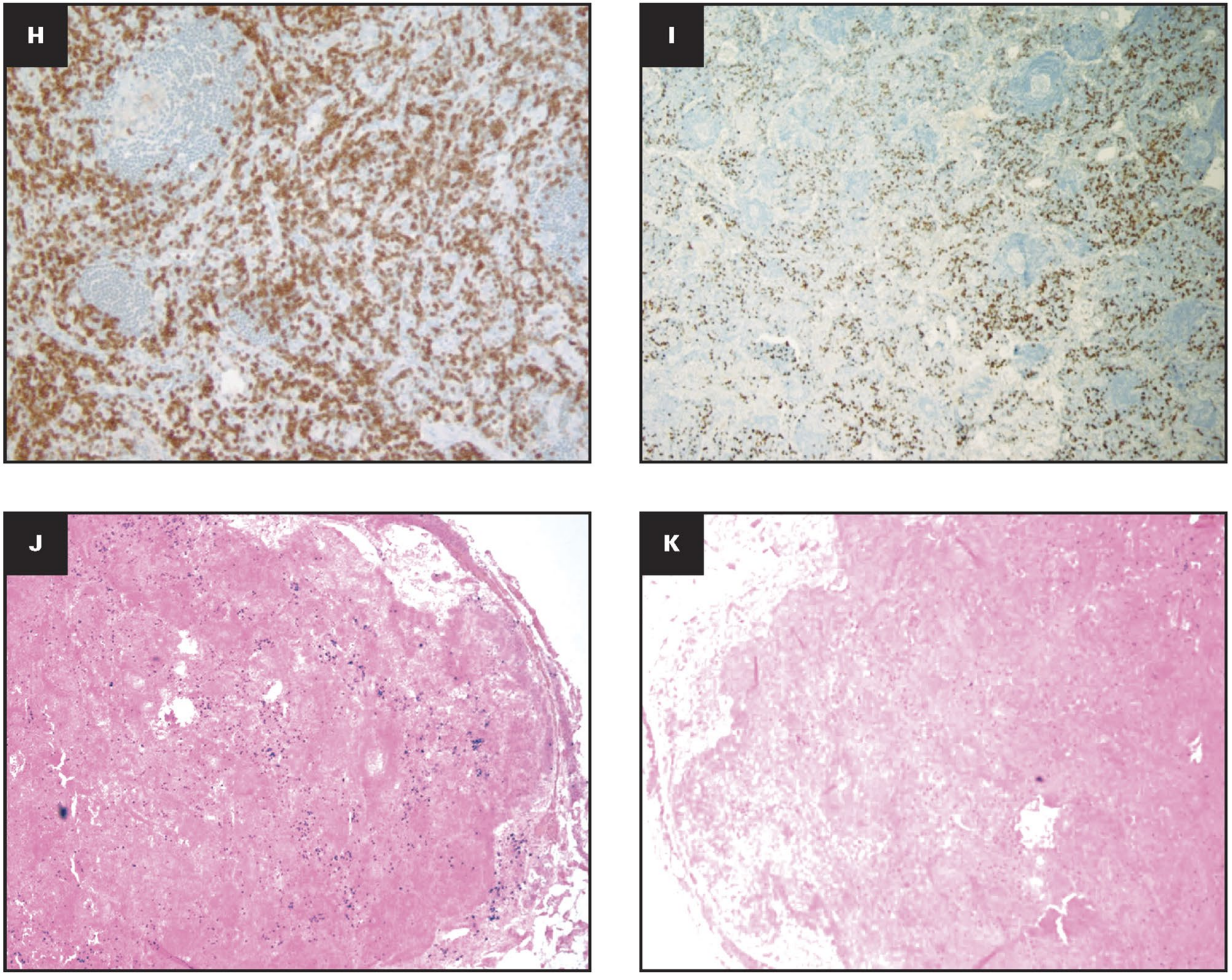
**Figure 8** Histopathologic characteristics of a bone marrow biopsy and an inguinal lymph node from an excisional biopsy. Case 2. **(A, B)** Bone marrow biopsy from an outside hospital displaying diffuse, prominent fibrosis and megakaryocytic hyperplasia with dyspoiesis (HE stain; **A**, 10x magnification; **B**, 40x magnification); **(C)** Moderate bone marrow fibrosis (reticulin stain in 20x magnification); **(D)** lymph node with partially distorted architecture, with scattered atretic follicles throughout the cortex and medulla (HE stain 2× magnification, grade 3 atretic follicles); **(E)** germinal centers of the follicles with lymphoid depletion; HE stain in 4x magnification (arrow, grade 3 follicular dendritic cell prominence); **(F)** lymphocyte-depleted interfollicular regions with numerous high endothelial venules and a subset showing sclerotic/hyalinized walls; HE stain in 4x magnification (grade 3 vascular hyperplasia); **(G)** ­Immunostain CD20-positive B lymphocytes highlighting atretic follicles in 4x magnification; **(H)** Prominent interfollicular immunostain CD3-positive T lymphocytes in 10x magnification; **(I)** Immunostain CD138-positive interfollicular plasma cells in 2x magification; **(J, K)** κ and λ light chains (in situ hybridization) in 2x magnification. HE, hematoxylin and eosin.

Laboratory findings were clinically significant for anemia (hemoglobin = 9.1 g/dL), mild leukocytosis (white blood cell count = 10.79 ×10^9^/L), thrombocytopenia (platelet count = 116 ×10^9^/L), abnormal kidney function (serum urea nitrogen = 49 mg/dL, creatinine = 1.6 mg/dL), increased CRP (135 mg/L), mildly elevated lactate dehydrogenase (624 U/L), increased ferritin (689 ng/mL), and increased IL-6 level (95 pg/mL). Lupus anticoagulant was falsely positive, and immunoglobulins were within normal limits. Specific serologic tests for SLE were negative. The morphology of the lymph node suggested a diagnosis of “Castleman lymphadenopathy of the hypervascular type,” and these findings, in addition to the immunophenotypic, clinical, and radiologic findings of lymph nodes, were consistent with involvement by the iMCD-TAFRO variant, meeting the definite iMCD-TAFRO criteria per the international definition.

In view of the severity of the illness, treatment was initiated with siltuximab 11 mg/kg, followed by 2 doses of rituximab and combination chemotherapy (bortezomib, dexamethasone, cyclophosphamide, and etoposide). Treatment was then continued with siltuximab administered at 11 mg/kg every 3 weeks. The patient’s blood count and laboratory results recovered; radiologically, there was no evidence of worsening lymphadenopathy, and some of the lymph nodes decreased in size. The patient received continuous treatment with siltuximab and achieved complete remission. She is presently on siltuximab 11 mg/kg every 3 weeks and more than 1 year after diagnosis is clinically well, with no evidence of enlarged lymphadenopathy or other abnormal laboratory findings suggestive of CD.

## CONCLUSION

The diagnosis of iMCD can be challenging due to disease presentation that overlaps with other causes of lymphadenopathy (such as infections, autoimmune disease, and malignancy) and a lack of specific disease biomarkers. An accurate diagnosis requires a multidisciplinary team of hematologists, pathologists/hematopathologists, and other specialists. By adding quantitative guidance to grade the histopathological features of iMCD, our harmonized system has the potential to help physicians accurately diagnose iMCD and distinguish morphologic mimicry, which is key to determine appropriate treatment and improve patient outcomes.

## Data Availability

No new data were generated or analysed in support of this research.
