# Correction to: Elucidating the grading intricacies of idiopathic multicentric Castleman disease histopathology: a pathologist’s perspective

**DOI:** 10.1093/ajcp/aqag064

**Published:** 2026-05-26

This is a correction to: Daisy V Alapat, Payam Etebari, Frits Van Rhee, Elucidating the grading intricacies of idiopathic multicentric Castleman disease histopathology: a pathologist’s perspective, *American Journal of Clinical Pathology*, Volume 165, Issue 3, March 2026, https://doi.org/10.1093/ajcp/aqaf134

The following changes have been made to the originally published paper.

The label on the third image in Figure 4 has been corrected to read: “Plasmacytic pathology” instead of: “Hyaline vascular (or hypervascular) pathology”. Figure 4 should read:

**Figure:**
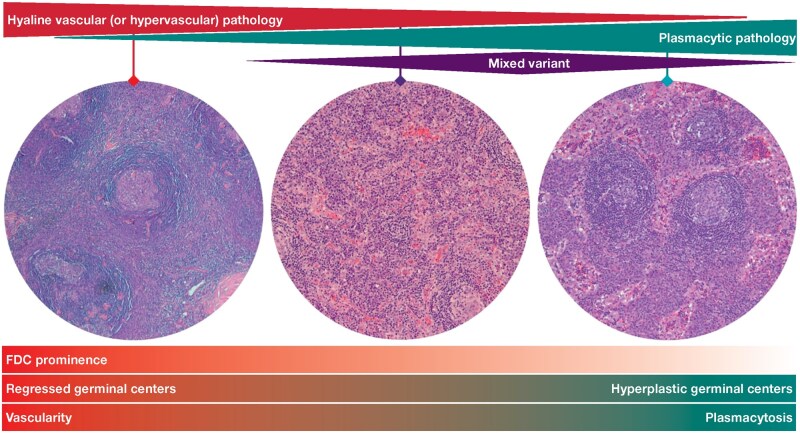


instead of:

**Figure:**
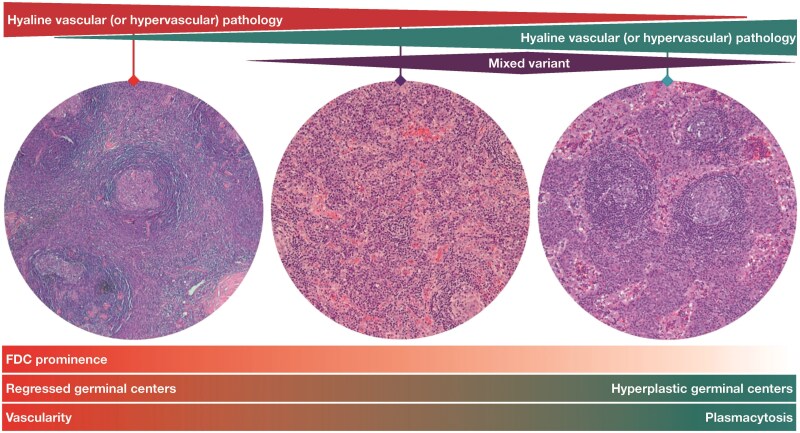


In the digital version of the paper, panels were missing for Figures 7 and 8. In Figure 7, panels G, H, I missing; in Figure 8, panels H, I, J, K were missing. These panels have now been supplied to the paper so that they appear, and agree with their respective legends, in both versions.

**Figure 7:**
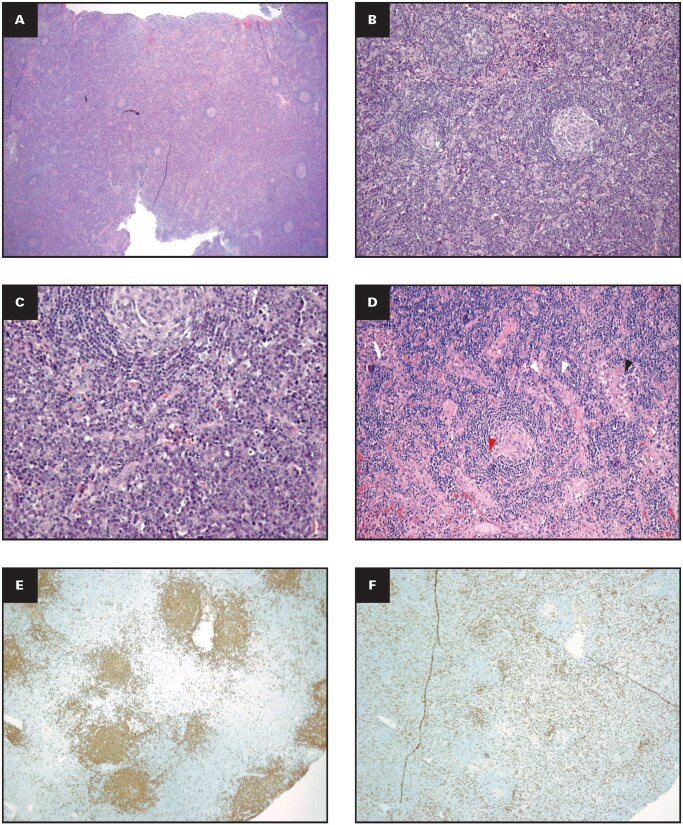

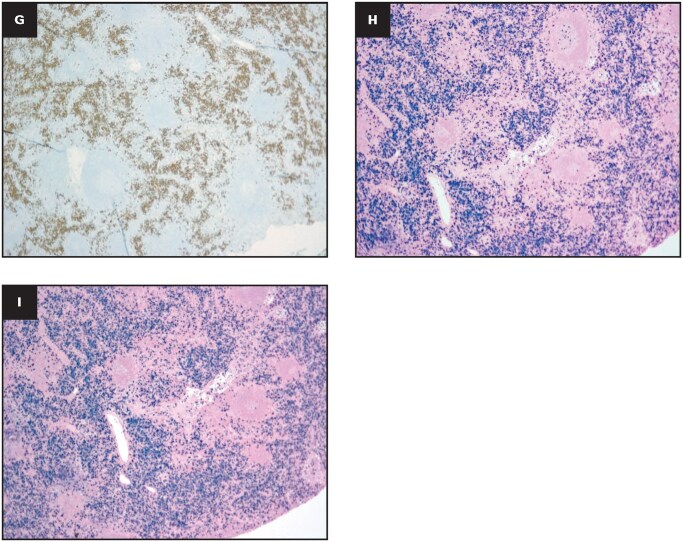
Histopathologic characteristics of a cervical lymph node from excisional biopsy (Case 1). **(A)** Lymph node with abnormal architecture with polymorphic follicles, with a predominance of hyperplastic germinal centers (grade 2 hyperplastic follicles; H&E, 2× magnification); **(B)** germinal centers with expanded mantle zones, with concentric layering and hyaline deposits (grade 2 follicular dendritic cell prominence; H&E, 10× magnification); **(C)** interfollicular areas with large sheets of plasma cells (grade 3 plasmacytosis; H&E, 40× magnification); **(D)** lymph node (H&E, 10× magnification) highlighting increased vascularity (white arrowheads), regressed follicles with hyalinized vessel–penetrating germinal center (red arrowhead), and open sinuses with histiocytes (black arrowhead); (E) CD20 immunostain–positive B cells highlighting germinal centers (4× magnification); **(F)** CD3 immunostain–positive T lymphocytes (4× magnification); **(G)** CD138 immunostain–positive interfollicular plasma cells (2× magnification); **(H, I)** κ and λ light chains, respectively (in situ hybridization, 2× magnification).

**Figure 8:**
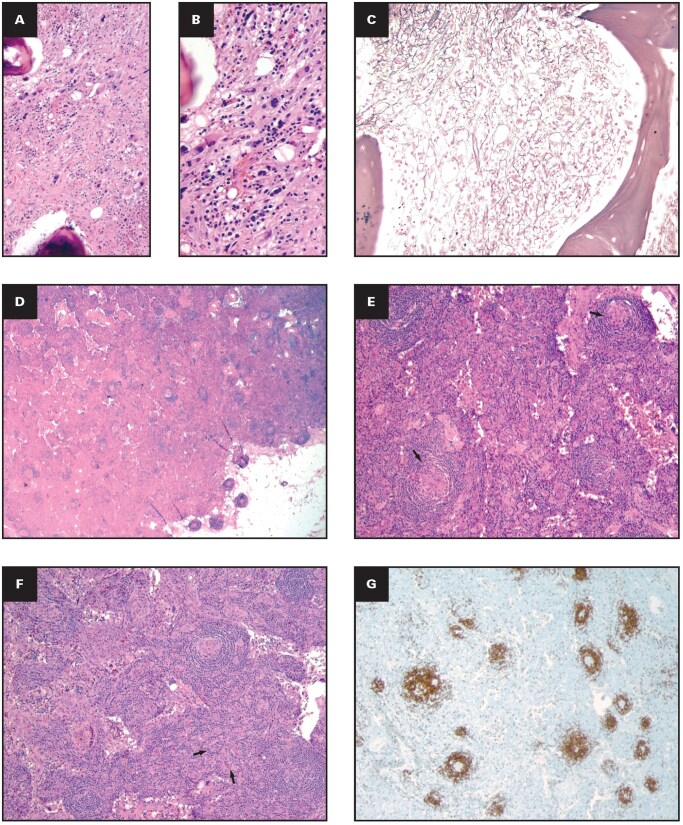

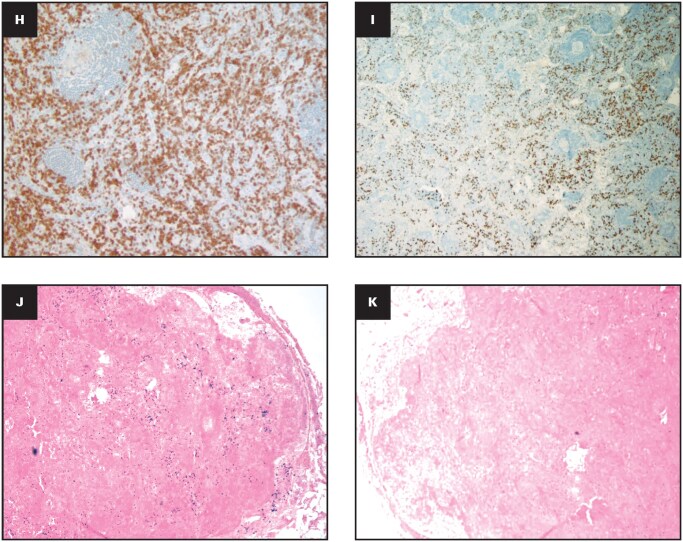
Histopathologic characteristics of a bone marrow biopsy and an inguinal lymph node from an excisional biopsy (Case 2). **(A, B)** Bone marrow biopsy from an outside hospital displaying diffuse, prominent fibrosis and megakaryocytic hyperplasia with dyspoiesis (H&E; **A**, 10× magnification; **B**, 40× magnification); **(C)** moderate bone marrow fibrosis (reticulin stain, 20× magnification); **(D)** lymph node with partially distorted architecture, having scattered atretic follicles throughout the cortex and medulla (grade 3 atretic follicles; H&E, 2× magnification); **(E)** germinal centers of the follicles with lymphoid depletion (arrows), grade 3 follicular dendritic cell prominence (H&E, 4× magnification); **(F)** lymphocyte-depleted interfollicular regions with numerous high endothelial venules and a subset showing sclerotic/hyalinized walls (arrows), grade 3 vascular hyperplasia (H&E, 4× magnification); **(G)** CD20 immunostain–positive B lymphocytes highlighting atretic follicles (4× magnification); **(H)** prominent interfollicular CD3 immunostain–positive T lymphocytes (10× magnification); **(I)** CD138 immunostain–positive interfollicular plasma cells in 2× magnification; **(J, K)** κ and λ light chain in situ hybridization (2× magnification).

